# Transition from an adolescent to an adult eating disorder treatment centre: A qualitative investigation of the experience of inpatients with anorexia nervosa and their carers using interpretative phenomenological analysis

**DOI:** 10.1002/erv.3030

**Published:** 2023-09-01

**Authors:** Elisabetta Scanferla, Sabrina Seryer, Bernard Pachoud, Philip Gorwood

**Affiliations:** ^1^ Clinique des Maladies Mentales et de l’Encéphale Hôpital Sainte‐Anne GHU Paris Psychiatrie et Neurosciences Paris France; ^2^ Université Paris Cité, ED 450 Paris France; ^3^ Université Paris Cité Institut de Psychiatrie et Neurosciences de Paris (IPNP) INSERM Paris France

**Keywords:** adolescents, adults, anorexia nervosa, care, carers, eating disorders, meaning‐making process, subjective experience, transition

## Abstract

**Aims:**

To capture the subjective experience of eating disorder patients and their immediate family members in the transition between adolescent and adult treatment services and to explore how both groups make sense of this specific experience.

**Design:**

Qualitative study in the form of in‐depth interviews using interpretative phenomenological analysis.

**Settings:**

Participants were recruited from a university department of a large psychiatric hospital specialising in eating disorders between July 2021 and September 2022.

**Participants:**

A convenience sample of 18 participants was recruited, including 12 patients aged 19–30 years (*m* = 22.5, SD = 3.8) and six of their respective caregivers.

**Results:**

Four main themes were identified in relation to the participants' experience of transition to adult care: (1) the detailed description of the transition process, (2) the emotions associated with this experience, (3) the challenges encountered and (4) recommendations for improving the process. Two fundamental meaning‐making processes emerged: the feeling of being "lost in transition" and the opportunity to "become an adult". The results highlighted the factors that characterise this experience for patients and their families, and the need for practical and psychological support during the transition process.

**Conclusions:**

This study provides a unique insight into the experience of patients and their immediate family members regarding the transition from adolescent to adult care. It reveals the multidimensional impact of the transition experience and highlights the need for increased support for family members. These findings may provide new insights into interventions that promote successful transition and encourage rethinking the organisation of this crucial stage of the care pathway.

## Key points

- The transition phase between adolescent and adult care is a challenging experience for patients and carers, marked by difficulties in accessing care, lack of information, high levels of fear and a sense of cultural change in the way care is organised and delivered in both settings.
- Parents play a key role in accompanying patients through the transition process. They need to be more actively involved in the preparation and arrival phase in adult care facilities and receive more support from health professionals.
- Assessment of the transition process from the user's perspective and new interventions, covering both adolescent and adult services, are needed to support a successful transition, better adapted to the needs of young adults with severe forms of anorexia nervosa. These include recovery‐oriented personal therapies with a focus on long‐term goals, as well as tailored and flexible intensive day treatment that allows the pursuit of educational and vocational goals.

## 1 INTRODUCTION

Transition of care refers to the "intentional and planned process (…) of adolescents and young adults with chronic physical illnesses moving from a child‐centred to an adult‐centred health care system" (Blum et al., 1993). It also concerns young people with mental health problems“ (…) such as those suffering from depressive and anxiety disorders or eating disorders” (Babajide et al., 2020; Herpertz‐Dahlmann & Schmidt, 2023). Its primary objective is to ensure continuity of care throughout the life cycle (e.g., maintaining care over time, ensuring a relationship between patient and care team, providing information transfer, and meeting changing needs) (Hu et al., 2020). However, the difficulties associated with the transition from child and adolescent mental health services to adult mental health services have been widely reported (Islam et al., 2016; McGorry et al., 2007, 2022; Singh, 2009; Singh et al., 2021). This suggests that health care systems are not meeting the needs of young adults (Mulvale et al., 2019; Reneses et al., 2022).

For all eating disorders, the proportion of onset before the age of 18 is 48.1%. The average age of onset for anorexia nervosa is 17 years (Solmi et al., 2022). A high proportion of people with this disorder require continued specialised treatment (Herpertz‐Dahlmann et al., 2021) and there is likely to be a need to move from child and adolescent services to adult mental health services (Castagnini et al., 2016; Wade, 2023). McClelland and colleagues noted that one third of patients seen in a child and adolescent eating disorder service in the UK were receiving further mental health treatment in an adult eating disorder service (McClelland et al., 2020).

The transition process is a crucial part of the care pathway for young patients. They must navigate new care environments and providers (Mulvale et al., 2019) while experiencing major changes in biological, social and emotional processing (Crone & Dahl, 2012; Dahl, 2004). Therefore, a smooth transition, focused on the needs of individuals, is essential for patients to achieve the best possible health outcomes. However, the perspectives of patients and carers are poorly understood and often overlooked in the provision of care during the transition sequence (Herpertz‐Dahlmann, 2021; Sibeoni et al., 2017). In the qualitative literature to date, the transition process is perceived as a challenge for all parties involved, including: physicians (Stocker et al., 2022) and young patients and their parents (Dimitropoulos et al., 2015; Lockertsen et al., 2020, 2021). The end of care for children is regularly reported as an abrupt loss of support and is associated with difficulties in accessing adult services (Dimitropoulos et al., 2015; Wales et al., 2021, 2022). Patients described the transition as a challanging and traumatic experience characterised by feelings of distress and discontinuity of care (Lockertsen et al., 2020; Nadarajah et al., 2021; Treasure et al., 2021).

Similarly, relatives reported that transition was a period associated with discontinuity of care leading to deterioration and relapse in their relative's health status (Lockertsen et al., 2021). Patients and carers highlighted the lack of support before and during this transition, as well as the lack of communication between services, between services and patients, and between relatives and professionals (Lockertsen et al., 2021; Wales et al., 2022).

Little research has been done on how to improve the smoothness of the transition process to different adult care settings for young patients with eating disorders. This research is needed to develop guidelines for managing the transition to adulthood for these patients (Reale & Bonati, 2015; Royal College of Psychiatrists, 2017; Treasure et al., 2005; Winston et al., 2012).

Despite the existence of qualitative findings on subjective experiences of transition, how patients and their carers make sense of these experiences, patterns of meaning‐making (e.g. the subjective construction that each participant may made of his/her experience of transition [Larkin, 2009]) and their implications for engagement in the transition process, remain largely unexplored (Herpertz‐Dahlmann & Schmidt, 2023).

The aim of our qualitative study was to capture the transition experiences of young people suffering from eating disorders who had been transferred from paediatric care to adult specialist hospital services, and their families, and to identify how they made sense of the subjective experience of transition. The objective aim was to generate new shared perspectives on this process of care by combining their views, and to retrospectively characterise the challenges, facilitators and needs that emerged during this key sequence of the care pathway.

## 2 METHOD

### 2.1 Participants and recruitment

Patients and their informal caregivers were assessed for eligibility in a university inpatient unit specialising in eating disorders at the *Paris Psychiatry and Neurosciences* University Hospital Group between September 2021 and June 2022.

The inclusion criteria for patients were (1) age ≥18 years at the time of the study; (2) a *DSM‐5* diagnosis of anorexia nervosa and/or bulimia nervosa (American Psychiatric Association, 2013); (3) admitted to an inpatient eating disorder unit; (4) received treatment for their eating disorder in a paediatric setting within the past 10 years. Exclusion criteria were (1) severe cognitive impairment precluding participation in the study, as determined by the investigators; (2) lack of proficiency in French to participate in the research interviews. All eligible patients admitted consecutively for intensive treatment in the specialised unit were offered to participate in the study. The recruitment rate was 100%.

Patients were also offered to designate a family member to participate in the study. The exclusion criteria were: (1) severe cognitive impairment preventing participation in the study, as determined by the investigators; (2) lack of French language skills to participate in the research interviews. Seven of the 12 patients included provided the name of an ascendant who was contacted. One ascendant refused to participate, 6 accepted and were invited to the interview.

All participants signed a research participation agreement.

### 2.2 Data collection and analysis procedures

In‐depth, semi‐structured qualitative interviews were conducted in person for patients, and by telephone for family members, by a research psychologist who did not know the participants prior to the study. She had experience with qualitative interview methods for over 5 years. Each participant was interviewed separately. Although the interviewers followed an interview guide, participants were encouraged to express themselves freely. This guide included questions about the experience of the transition from adolescent to adult eating disorder treatment and the emotional state of this experience. It was written by the first author (ES) in collaboration with two authors (BP and PG). The final version of the guide is available as a supplementary information file (Additional file 1). The average duration of the interviews was 55 min. Data collection and analysis were conducted simultaneously.

The interviews were audio recorded and transcribed verbatim. All interviewees were given the opportunity to review their transcripts and provide feedback. They were then anonymised by removing all identifying information. The original French transcripts were translated into English. A bilingual French‐English researcher checked that the translation of the English verbatim was consistent with the meaning of the French source texts.

Interpretative phenomenological analysis (IPA) was chosen to explore the content of the interviews (Smith & Shinebourne, 2012). This method of analysis was developed to understand the complex system of meanings attached to a single, subjective and highly intimate phenomenon (Larkin, 2009). The experience of transition in the care pathway is made up of memories, impressions and emotions, and IPA allows access to the subjective construction that each participant may or may not have made, remaining attentive to the subtleties of each experience, in all its complexity (Larkin, 2009). Authors 1 and 2 (ES and SS) conducted the analysis following IPA principles and identified themes in the discourse. Each interview was read several times by the first author to obtain a holistic view of the participant's experience. Data saturation was discussed by authors 1 and 2. All transcripts were coded by the lead researcher (ES) and 50% of these transcripts were double coded by the second author (SS) to ensure accuracy. The main themes of the speech and the links between themes were identified by the first author. Finally, an interpretive account was produced to highlight and analyse the experience through experiential themes and meaning‐making processes, defined as the method by which people attempt to make sense of their experiences.

Throughout the process, we were careful to respect the criteria of scientific rigour established by the qualitative analysis (Fossey et al., 2002). The data were processed and analysed using NVivo 12.0 software developed by QSR International. The report of our study meets the COREQ (Consolidated criteria for Reporting Qualitative Research) checklist, which provides criteria for ensuring the quality of qualitative data (Tong et al., 2007).

## 3 RESULTS

### 3.1 The participants

Eighteen participants were recruited for the study: 12 patients and 6 carers (4 mothers and 2 fathers), who formed 6 dyads with their relatives. A total of 18 interviews were conducted. The patients were all women aged between 19 and 30 years (mean = 22.5, SD = 3.82), with a body mass index between 13.0 and 18.5 (mean = 15.7, SD = 1.75) (Table 1). The patients included suffered from anorexia nervosa (12): restrictive subtype (9) and binge‐eating/purging subtype (3).

**TABLE 1:** Characteristics of 12 inpatients with eating disorders, and 6 of their caregivers (parents), who moved from an adolescent ward to an adult ward.

Participant	Type	Age (years)	Diagnosis	BMI	Duration of illness (months)
P1	F	24	AN r	15.4	84
P2	F	20	AN r	13.0	36
P3	F	28	AN p	13.8	108
P4	F	26	AN r	16.8	72
P5	F	20	AN r	14.1	24
P6	F	21	AN r	15.8	36
P7	F	19	AN r	16.8	24
P8	F	19	AN r	18.2	12
P9	F	24	AN p	18.5	60
P10	F	20	AN p	16.9	36
P11	F	19	AN r	14.2	10
P12	F	30	AN r	15.9	131
C1	F	40–50			
C2	F	50–60			
C3	F	40–50			
C4	F	>60			
C5	M	30–40			
C6	M	50–60			

*Note*: "P" stands for "patient", "C" stands for "caregiver"; "F" stands for female, "M" stands for male; "AN r" stands for restrictive anorexia nervosa, "AN p" stands for bulimia nervosa/purgation.

Abbreviation: BMI, body mass index.

The mean time difference between the transition, and the qualitative interview for each participant was 15 months. The median was 4.5 months.

### 3.2 Analysis of the main themes

Four main themes emerged from the analysis: (1) Description of the transition process; (2) Emotions experienced; (3) Challenges of the transition; (4) Facilitators and recommendations to improve the process (Table 2).

**TABLE 2:** Main themes and sub‐themes.

Main themes	Subtopics
Description of the transition process characteristics	Modalities and management (age limit, fluidity, new start…).
	Role of informal carers (practical and emotional support). The arrival in adult care services, a cultural change (rules of the environment, harshness…).
Emotions experiences	Patients' feelings (fear, overwhelm, abandonment…). Feelings of the carers (destabilisation, helplessness, powerlessness….).
Challenges of transition	Delayed access to adult care (adverse effects…). Lack of support from health care providers (unprepared transition…).
Facilitators and recommendations to improve the process	Continuity of care. Supporting the achievement of personal life goals (professional and personal). Involvement of carers (emotional and operational support). Being accompanied and welcomed in the adult care facility.

In the section below, the letter "P" at the end of the sentence refers to a patient's verbatim and the letter "C" to a carer's verbatim.

#### 3.2.1 Description of the transition process characteristics

##### Modalities and management

The end of the adolescent care sequence is perceived by a large majority of patients and families as a time of disruption and hasty discharge from adolescent services:

After the last hospitalisation it was over, there was nothing more, no more contact [*with the adolescent care team*] (P12)

On the other hand, some young adults saw the move to an adult service as an opportunity to gain a new perspective on their care, which could be enhanced by the opportunities to interact with new health professionals, treatments and environments:

I was no longer satisfied with the care I was receiving [*adolescent care*]; it was time to move on… (P9)

##### Role of informal carers

Patients' families were described as having a central role in the transition process. In all interviews, this role was mainly characterised by two dimensions:

‐ researching and finding specialised adult facilities, using personal resources (personal networks, Internet and printed resources…)

My grandmother did a lot of research on the internet about adult places and she found this one [*the adult unit*] … (P8)

‐ helping their relatives with administrative procedures.

If young people are not followed, or even pushed, by their parents, it doesn't work. The children can't manage on their own… she [*her daughter*] can't manage it. It's absolutely impossible. (C4)

Due to the shortage of beds in adult specialist services, participants unanimously expressed their difficulties in accessing care in a timely manner and the importance of personal relationships.

It's too complicated to get into a specialist adult service, you have to have personal connections in the care sector to find a place. (P5)

In particular, one parent pointed out that this lack of specialist facilities could potentially create inequalities in access to care.

Parents who manage to get their children into a specialist adult service are more aware, have more knowledge, more contacts… which in my opinion creates an inequality in access to care! (C4)

Finally, several patients stressed how important it had been for them to receive help from their parents to make informed decisions about the transition process and their treatment, decisions that they were expected to make alone as adults.

They wanted to give me antidepressants, I had to make the decision, but I was lost…, so I discussed it with my mother, and we decided together. (P4)

##### The arrival in adult care services, a cultural change

The vast majority of participants interviewed, both patients and carers, described the move as a sudden cultural change that forced them to adapt to a philosophy and therapeutic model of care that was very different from what they had experienced in the paediatric setting. In particular, they pointed out that in the adult setting the rules were much stricter and the atmosphere colder than the family atmosphere in the child and adolescent wards.

Here [*adult service*] it's not the same, it's hard, a bit military. So yes, it's much tighter and harder in fact. (C8)

However, three patients highlighted the positive effects of a more restrictive environment.

Here [*adult ward*] it's stricter than in the paediatric ward, but here I can't do silly things, like taking pouring soup in the sink… These strict rules is precisely to protect you from doing silly things (P7).

#### 3.2.2 Emotions experienced

##### Patients' feelings during the transition

Most patients and their relatives spontaneously highlighted the psychological impact of the transition from paediatric to adult care and described it as a difficult and destabilising experience. Specifically, they reported feeling unprepared for the transition, having to leave paediatric professionals they knew and had close ties with, and feeling uncomfortable about having to build relationships with and trust the new care staff. These experiences were associated with fear, overwhelm and a sense of abandonment, which were still vivid memories at the time of the interview.

I was really scared… Because I was 18, I didn't understand why they [*adolescent facility*] didn't want me anymore… (…) going from an adolescent to an adult environment can be very, very scary. (P10)

Other intense emotions mentioned by the majority of participants were high levels of anxiety and the feeling of being out of step with other patients. These emotions are linked to several variants, namely the many rules governing the adult environment and the fear of being rejected by other patients.

When I arrived in an adult service, it was a big difference and I felt very stressed: "Will I be welcomed? "Will I not be left out or isolated? I was afraid to be with adults only (P6)

An ambivalence emerged in the interview data, as some patients, while expressing fear, also stressed that they felt very "lucky" to have been admitted to a specialised facility, given the lack of beds, and that they considered this an important step forward:

I am lucky to be here [*adult setting*], there are few places. (P8)

Four patients spoke of the emotional support and encouragement to cope with negative emotions provided by their relatives throughout the transition period, from the preparation phase for childcare to the arrival in the adult facility.

My parents helped me a lot during my transition; they always supported me and encouraged me to pursue my treatments (P9).

##### Feelings of carers during transition

The transition period also caused emotional distress among family members who expressed feelings of helplessness, powerlessness and distress at the difficulty of finding a place in an adult institution, while witnessing their child's deteriorating health.

We didn't really know how things would end with her. And then we didn't really know what would happen next. It was very destabilising (C4)

In addition, carers reported similar feelings related to the perceived lack of support from adolescent and adult care teams during the transition process.

I didn't know what it was like [*adult setting*], that I had no picture of the place, I felt helpless… I couldn't try to reason with her and say, "Honey, I understand it's hard, but your room's not bad after all. Everything will be all right"! (C2)

#### 3.2.3 The challenges of transition

##### Delayed access to adult care

The time taken for admission to the AMHS was described as “not very long" by one patient (P9) and very long by most patients and relatives. This was identified as the main difficulty in the admission process:

What really bothered me was the delay. In fact, the delay was so long that… I had moments when I almost died (P8).

Half of the patients reported negative effects of waiting to access adult specialist services, including increased symptoms, overall deterioration of their health, decreased motivation to stay in the care system and disruption of care, among other effects.

For me it was too long because I didn't have any follow‐up and I relapsed. In fact, I found it very long and difficult to stay at home alone for months… It seemed like forever. It was very scary. (P1)

##### Lack of support from health care providers

Patients and carers spontaneously reported a gap between their expectations of the role of professionals in initiating and supporting the transition to adulthood and the reality of the experience. They emphasised the withdrawal of health professionals and the need for the personal involvement of patients and informal carers to bridge this gap:

With no information from the paediatric ward, I went to my GP (…), but he didn't really know how to help me, so I looked on the internet… (P6)

Young adults also highlighted the perceived lack of expertise of health professionals during the transition process, as well as the lack of communication between them.

My paediatrician (…) was completely lost (…), he didn't really know (…) where to turn (P6).

Parents and patients also described a lack of knowledge among child and adolescent care staff about adult eating disorder services, how to identify them, how to access them and how to organise care in these facilities, and thus a poor overall ability to coordinate care pathways with these services.

We had no information other than the advice to look for a place in adult care… (C5)

It was also felt that there was no real link between the different services involved in the patient's treatment, and that there was insufficient communication between them.

Certainly they [*the youth and adult services*] don't talk to each other. (C6)

#### 3.2.4 Facilitators and recommendations to improve the process

##### Continuity of care

In terms of the most useful factors in the transition process, more than half of the participants stressed the importance of support from health professionals.

My therapist helped me much more by e‐mail, by phone… trying to accompany me every step of the way, if I needed help… (P10)

In addition, a smooth and personalised transition process, carefully planned and prepared by adolescent services, was described as a key facilitating factor.

The problem is that you are one person, the system has to work for us and not the other way round… (P2)

##### Supporting the achievement of personal life goals

Intrinsic and extrinsic motivation, a better understanding of one's disorder and the desire to move forward in life were the main sub‐themes that emerged from the analysis of the qualitative data.

I felt ready to come here [*adult setting*] … it's a question of personal motivation, you have to be ready (P1)

For the majority of participants, the desire to have a rich social life, to succeed academically and to progress in their professional life are motivating factors for seeking professional help and remaining in the care system.

What motivated me the most was that I couldn't follow my studies properly. (…) and I really wanted to… pursue the goals I had set for myself, both academically and professionally… (P10)

Two family members emphasised that the possibility of flexible care arrangements was crucial, as it allowed them to pursue studies or professional activities.

My daughter is going to university and we didn't want her to spend several months in hospital… She already has health problems, but if she has school problems on top of that… I think it adds more complexities to the illness. (C4)

Overall, the interviews showed that intrinsic and extrinsic motivation and reinforcement of personal life goals were key drivers of action, enabling patients to remain engaged in care with their families.

##### Involvement of carers

Most patients emphasised the essential role of family members in compensating for the lack of professional support, in helping them make decisions about their treatment and care, and in providing emotional comfort.

Whatever our age, we also need people we trust, love and appreciate. We know they will help and support us through this challenge (P6)

##### Being accompanied and welcomed in the adult care facility

Half of the parents interviewed stressed the importance of better communication between youth and adult care services and emphasised the value of appointing a health professional as the key person to interface between the different care settings.

It would be good if someone in the team was responsible for connecting the dots… connecting the care teams. It would have helped my daughter a lot, and me too! (C5)

The need for more information before entering an adult care facility to better understand its characteristics was unanimously mentioned by patients or their relatives.

What I would have liked was to help my parents visualise things… like the room where we have meals. It would have brought us closer and maybe they would have been less worried about me at first… (P8)

Finally, patients and their relatives stressed the importance of feeling fully supported by the whole health care team in adult facilities. They particularly emphasised the moment of arrival and welcome on the ward, stressing that feeling welcomed gives hope to families and relatives and encourages young patients to remain involved in care.

It's very, very important to be welcomed by the health care staff, it changes everything… (P1)

### 3.3 Interpretative account

The interpretive narrative, based on the main themes described above, provided in‐depth insight into how participants made sense of their experience of the transition process and highlighted the convergences and divergences between the experiences of participants. Despite some specificities, all of them experienced the transition as a more or less difficult sequence in which family support was crucial.

#### "Lost in transition"

Some participants expressed great difficulty in making sense of the transition experience. Two dimensions emerged in the search for meaning. On the one hand, the difficulty of finding a "place" in a specialised adult facility, both in terms of lack of knowledge of where to go (identification of the facility) and limited treatment offers in specialised facilities due to lack of capacity. On the other hand, negative emotions resulted from the lack of adequate support from health care providers, due to the perception of unpreparedness. Therefore, it can be difficult for young adults in transition to become responsible for their own care. A typical example of the feelings generated by the gap between the support patients expected and received is illustrated below:

I was given the booklet [*about the adult facility*], so I had it for four months, I was able to read it, reread it and reread it again…. it allowed me not to arrive here completely lost… So that's why I was really attached to the information in that booklet, but when you come in and it's not the case at all, it's a bit weird. (P8)

Finally, the feeling of being 'lost’ and the difficulty in making sense of the transition experience also emerged from the carers who felt rejected, '*outside the care*’, without being taught how to effectively support their relatives and participate in decisions about their care as adults. As a result, all parent participants pleaded to be considered an integral part of their loved ones' care team.

It was very frustrating and destabilising not to be involved in our child's care'(…). I think it's a shame because I could have helped if I had been told how to do it… (C5)

#### Moving towards adulthood

Despite the difficulties associated with the transition, some patients highlighted the positive effects of a 'new’ start in adult care and the need for a strict framework to help them make the most of this new stage in their care pathway, seeing the transition as an opportunity to move into adulthood. Patients highlighted that being empowered to make care decisions and focus on personal life goals was empowering

In the "adult department" we were told: "You will be more active in your care, etc. (…). It was quite motivating to tell yourself that you were becoming an 'adult', that you had no longer passively accept decisions made by others. (P5)

Overall, the findings converge and highlight the difficult dimension of the transition process to adult care services and the key developments needed to ensure a more effective and smooth transition.

## 4 DISCUSSION

We explored the experience of transition from adolescent to adult care for young adults with eating disorders and their parents. Four main themes were identified, encompassing (1) a detailed description of the transition process, (2) the emotions associated with this experience, (3) the challenges encountered, and (4) recommendations for improving the process, including the desire for greater specialist support and inclusion of carers in the treatment process. Analysis of the main themes revealed an interpretive narrative that provided insight into the meanings given to the experience of the transition. Despite some group specifics, participants shared an overall distressing and disjointed experience and a lack of support throughout the transition process. For many patients and parents, these feelings and impressions were dominant and hindered their meaning‐making process; for some patients, the transition, despite its challenges, was experienced as an opportunity to move towards autonomy in their care and in their lives.

Our results are in line with previous studies on the process of transition to adult care for eating disorders. In particular, they stress the need to organise discussions on the transfer between facilities for adolescents and those for adults prior to the transition, as well as a period of joint work; to develop individualised transition plans involving the young adult's parents; and to identify a liaison person to facilitate communication between the players involved throughout the care pathway. With regard to improving the organisational dimension of the process, several studies have suggested the need to focus on early identification of young people likely to need transfer, early preparation for transition involving child and adult care teams, individualised transition plans (Winston et al., 2023) and early intervention (McClelland et al., 2018). Previous research has also highlighted the importance of a period of overlapping joint working with adult services during the transition phase to manage the handover and help the patient meet key staff in the new service (Royal College of Psychiatrists, 2017) as well as the need to appoint a dedicated health professional, a transition coordinator (Winston et al., 2023) or a 'case manager’ (Foà et al., 2019) to act as a liaison person to facilitate communication between relevant stakeholders throughout the process (Nadarajah et al., 2021; Wales et al., 2021).

With regard to the need to support patients and parents during the transition process, other studies have raised key aspects of the role of health professionals, such as: guidance and assistance in accessing adult services (Dimitropoulos et al., 2015) and facilitating opportunities for young patients to maintain close relationships with their carers (e.g. ensuring that young people and their families can spend enough "quality time" in welcoming and safe spaces; offering programmes to develop communication skills within the family…) (Colton & Pistrang, 2004; Sibeoni et al., 2017; Winter 2015). Providing up‐to‐date information on available adult care facilities (Mooney et al., 2023) and their organisation has also been proposed (Paul et al., 2015). As highlighted in the participants' accounts, the reliability of the information provided is crucial at this particularly stressful time in the care journey. These specific aspects are possible areas for improvement.

Concerning the emotional dimension of the transition experience, several qualitative studies have found that late access to adult care is associated with acute psychological distress (feelings of fear and helplessness) and has a negative impact on health outcomes and recovery prognosis (Lockertsen et al., 2021; Treasure et al., 2020). Our findings further reinforce the importance of human relationships and the need for experienced 'sensitive and developmentally aware staff’ (Hay et al., 2014; Herpertz et al., 2019; Lock & La Via, 2015), skilled in establishing and maintaining the therapeutic alliance with young adults and their carers during the transition process (Paul et al., 2015).

In terms of clinical and organisational implications, our results suggest that two new strategies should be considered and further explored to facilitate a successful transition to adult care. The first proposal should be to focus on the long‐term personal goals of young adults at an early stage of acute care, even if patients present with severe symptoms (Touyz et al., 2013). In clinical practice, this could encourage the implementation of recovery‐oriented personal therapies related to hope, purpose and meaning in life (Leamy et al., 2011). The recovery model has recently been proposed as a way of adding value to current practice in anorexia nervosa, which focuses on personal empowerment and improving quality of life (Dawson et al., 2014; Wetzler et al., 2020). Thus, quality of life and the achievement of personal goals, rather than being an outcome of recovery, can become the instrument for achieving it (Hay et al., 2012; Mitchison et al., 2016). This type of intervention should be extended to both adolescent and adult services to ensure consistency in the therapeutic approach and to form a backbone around which to consolidate the transition process.

In the same vein, it may be worth developing personalised treatment programmes for young adults with severe forms of anorexia nervosa that can be more easily combined with the parallel pursuit of academic goals rather than traditional hospital programmes (Allen et al., 2023). Being able to receive specialised care without having to give up social and academic activities for several months was reported as a major motivating factor facilitating the continuity of care (Davey et al., 2023; Zipfel et al., 2023.). Tailored, flexible intensive day treatment programmes appears to be a promising treatment option that allows for the maintenance of greater social networks and better addresses the needs of emerging adults (Herpertz‐Dahlmann, 2021; Potterton et al., 2020).

Finally, future studies should also consider the development of standardised patient‐reported outcome and experience measures (PROMs and PREMs) to assess the transition to adult care. If used routinely in clinical practice, they could be key indicators of quality of care at all stages of the process, and provide opportunities for benchmarking and sharing of best practices across mental health systems (Scanferla et al., 2023). This would help to establish guidelines to better manage the transition to adulthood of young patients with eating disorders.

### 4.1 Strengths and limitations

This study has several limitations. The first is related to the representativeness of the sample. The participants were recruited from a list of inpatients in a single specialised adult institution. Therefore, the generalisability of our results may be questioned. Future research should include systematic recruitment of participants from several adult specialist wards providing different types of care. A second limitation is that the number of parents included was smaller than the number of patients, as only 6 patient‐parent dyads were included. However, we observed that data on the parents' experience was saturated at the sixth interview, whereas it was reached at the 11^th^ interview on the patients' experience. A third limitation was the composition of the research team, as the investigator worked in the ward where the patients were recruited. However, the researcher was not involved in inpatient care. A fourth limitation is related to the retrospective design of the research, which could have induced a reconstruction bias. An interesting direction for future research could be to design a longitudinal study, exploring the experience of patients and carers at three different time points: before transition, during the process and shortly after admission to adult services. This would capture variations in participants' experiences and provide a deeper insight into the trajectories of the subjective meaning‐making process.

Despite these limitations, this research provides insight into the personal experience of the transition process from the combined perspective of young adults and their parents. It suggests that although the transition process is a critical sequence where the risks of treatment discontinuation or relapse are high, this period, if properly prepared, may turn "into moments of care" (Stocker et al., 2022) and accompany the person holistically towards healthy maturation and adulthood.

## 5 CONCLUSION

This study highlights the challenges associated with the experience of transition from adolescent to adult specialist care, from the joint perspective of patients and their carers. It offers new insights into interventions that can support a successful transition process and encourages a rethinking of the organisation of care services based on the unique needs of the emerging adults.

## AUTHOR CONTRIBUTIONS

E.S., B.P. and P.G. participated in the design of the study. S.S. contributed to the coding of the interviews and the identification of discourse themes. P.G. had access to all study data and provided clinical interpretation of the results. E.S. draughted the manuscript. All authors reviewed, edited and confirmed their acceptance of the final submitted version.

## CONFLICT OF INTEREST STATEMENT

The authors do not declare anything.

## Supporting information

Supporting Information S1

## Data Availability

The datasets analysed in this study and which support its conclusions are available from the corresponding author upon reasonable request.
