# Supplementary material for: Transition from an adolescent to an adult eating disorder treatment centre: A qualitative investigation of the experience of inpatients with anorexia nervosa and their carers using interpretative phenomenological analysis
Source: Eur Eat Disord Rev. 2023 Sep 1;33(6):1162–74. doi: 10.1002/erv.3030 (PMC12547370; doi:10.1002/erv.3030)
Supplement: Supplementary file 1 — Supporting Information S1 [file ERV-33-1162-s001.docx]

**Additional file 1.** Semi-structured interview guide.

For patients:

1. Could you tell me about your care pathway?
2. Could you tell me about your experience of the transition process from adolescent to adult care services?
3. Could you tell me about factors that were barriers to this transition process?
4. Could you tell me about factors that facilitated this transition process?
5. Could you tell me about the place and role of your parent and family during this transition process?
6. What are your recommendations for improving this transition process?
7. Could you tell me about your arrival in a specialised adult care service?
8. What were your expectations when arriving in a specialised adult care service?

For their parents (carers):

1. Could you tell me about the care pathway of your loved one?

2. Could you tell me about your experience of the transition process from adolescent to adult care services of your loved one?

3. Could you tell me about the factors that were barriers to the transition process of your loved one?

4. Could you tell me about the factors that facilitated the transition process of your loved one?

5. Could you tell me about your role in the transition process of your loved one?

6. What are your recommendations for improving the transition process to a specialised adult care of young adults?

7. Could you tell me about your relative's arrival in a specialised adult care service of your loved one?
